# Brain Signature Predicts Negative Emotion in Individuals

**DOI:** 10.1371/journal.pbio.1002179

**Published:** 2015-06-22

**Authors:** Janelle Weaver

**Affiliations:** Freelance Science Writer, Carbondale, Colorado, United States of America

## Abstract

Emotions are central to our daily lives, but their neural basis is not well understood; a new study uses fMRI to identify a complex multisystem neural signature of negative emotion induced by viewing distressing photographs.

Emotions are central to our daily lives and are at the root of disorders such as anxiety, depression, drug abuse, and cardiovascular disease. The importance of understanding the emotional brain has motivated hundreds of neuroimaging studies in healthy humans and patients with psychological disorders. But there has been a pressing need to identify neural signatures that predict emotional experiences in individuals in order to translate brain-imaging data into clinical applications.

In a study published this week in *PLOS Biology*, Luke Chang and Tor Wager of the University of Colorado at Boulder and their collaborators used functional magnetic resonance imaging (fMRI) and machine-learning algorithms to identify a sensitive and specific brain signature that predicts the intensity of negative emotion in individuals [1]. Strikingly, this signature encompasses multiple widely distributed neural networks rather than a few individual emotion-related brain regions. According to the authors, the neural signature could be used to diagnose mental illness, predict responses to treatment, and guide the development of new brain-based therapies.

In the new study, the researchers used fMRI to measure brain activity in 183 individuals who viewed a sequence of 15 negative photographs and 15 neutral photographs and rated the emotional intensity of each image on a five-point scale. The negative photographs depicted bodily illness and injury, acts of aggression, members of hate groups, transportation accidents, and human waste, whereas the neutral photographs consisted of inanimate objects or nonevocative scenes (Fig 1).

**Fig 1:**
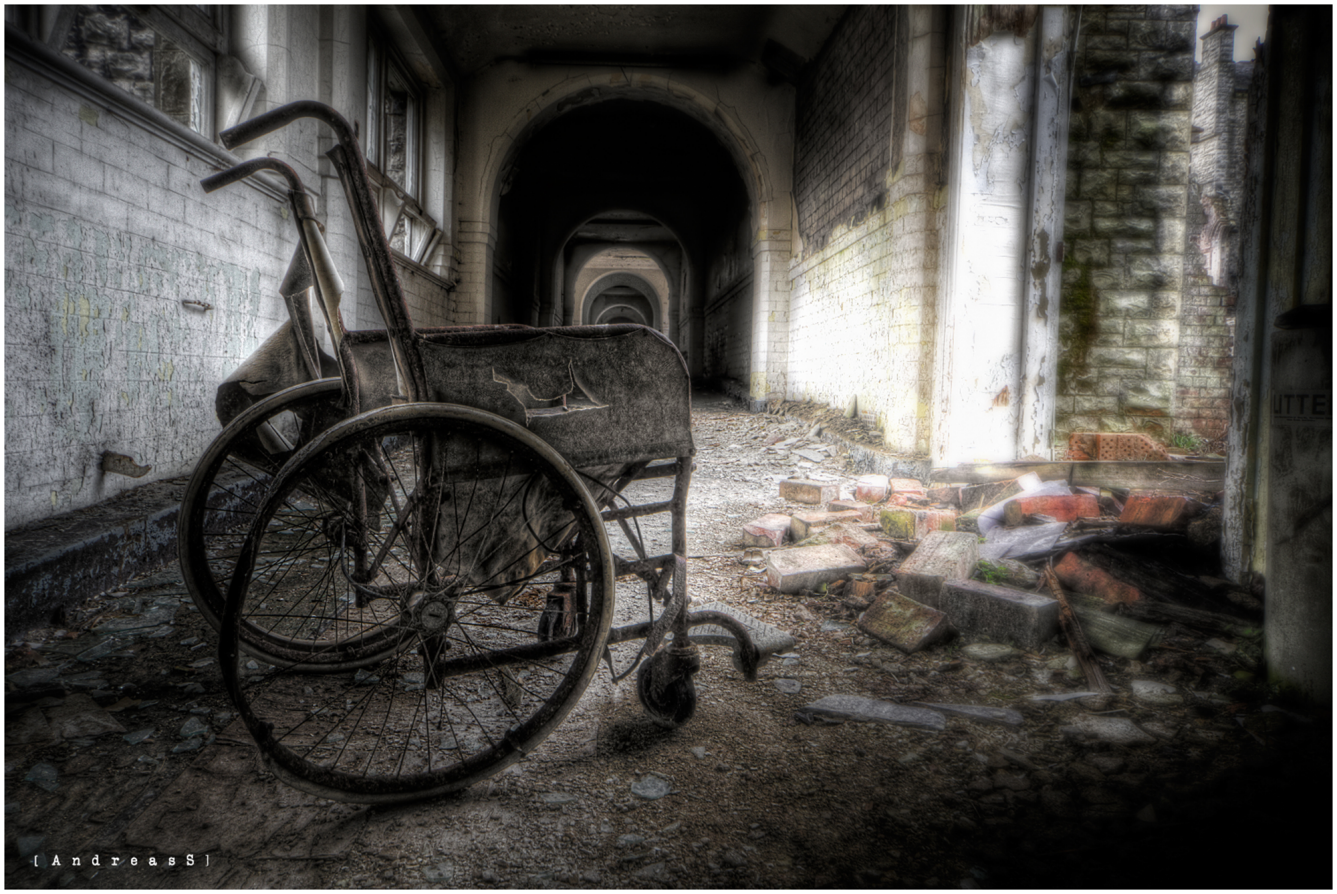
Luke Chang, Tor Wager, and colleagues used images similar to this to elicit negative emotions in the study participants while their brains were being scanned. *Image credit*: *AndreasS*, *Flickr*.

The researchers then used machine-learning algorithms to find global patterns of brain activity that best predicted participants’ ratings. This neural signature, called the picture-induced negative emotion signature (PINES), accurately predicted ratings of negative emotional experience in 94% of the participants. In more than 90% of the subjects, the PINES accurately distinguished between aversive pictures whose ratings were separated by two or more points on the five-point scale.

As expected, aversive pictures produced activity in brain regions typically associated with negative emotion, including the amygdala, anterior insula, and anterior cingulate cortex. However, activity in each of these regions was not strong enough to predict emotion ratings. Moreover, the PINES more accurately predicted emotion ratings than did activity in seven other previously reported neural networks associated with diverse functions such as rest, vision, attention, touch, and movement.

The researchers next used algorithms to separate the PINES brain regions into discrete subnetworks based on similar patterns of activity from trial to trial. They identified nine subnetworks involved in diverse functions such as vision, arousal, memory, social cognition, and action. Several of these subnetworks individually performed well at predicting emotion ratings, but all of them were less accurate than the PINES. Using a virtual lesion analysis, the researchers found that removing individual subnetworks from the PINES resulted in only negligible decreases in performance. The findings demonstrate that no specific subsystem is either necessary or sufficient for predicting negative emotion.

Finally, the researchers determined whether the PINES is specific to negative emotion and is not driven simply by general arousal. To do so, they compared the PINES to their previously reported neurologic pain signature (NPS)—a brain map that predicts the intensity of thermal pain. Similar to the PINES, the NPS was generated by a combination of fMRI and machine-learning algorithms. Comparison of the two signatures revealed that the experiences of negative emotion and pain are predicted by distinct patterns of neural activity. The PINES accurately predicted negative emotion but showed no response to increasing pain intensity. Conversely, the NPS responded robustly to increasing pain but showed no response to increasing negative emotion.

Taken together, the results suggest that the neural signature predicting negative emotion spans multiple brain systems and cannot be boiled down to a single network or a few brain regions that are traditionally associated with negative emotion. Therefore, the study calls into question theories that have treated emotion as an isolated experience that is localizable to a specific neural system. By providing an alternative, more nuanced model for the brain representation of emotion, the findings have broad implications for future attempts to identify biomarkers for mental health disorders.
